# Herpes zoster vaccine awareness and acceptance among adults in Saudi Arabia: a survey-based cross-sectional study

**DOI:** 10.1186/s40794-023-00202-z

**Published:** 2023-10-21

**Authors:** Sarah AlMuammar, Afaf Albogmi, Manar Alzahrani, Fai Alsharef, Raghad Aljohani, Teif Aljilani

**Affiliations:** 1 Family Medicine Department, Faculty of Medicine, King Abdulaziz University, Jeddah, Saudi Arabia; 2 College of Medicine, King Abdulaziz University, Jeddah, Saudi Arabia

**Keywords:** Herpes zoster, Shingles, Vaccine, Saudi Arabia, Awareness, Health literacy, Vaccine hesitancy

## Abstract

**Background:**

Herpes zoster (shingles) is caused by reactivation of the varicella-zoster virus. Despite the recommended herpes zoster vaccine for individuals aged ≥ 50 years, its uptake remains low in Saudi Arabia.

**Methods:**

This cross-sectional study assessed knowledge and awareness of herpes zoster and its vaccine in individuals aged ≥ 50 years in Saudi Arabia. Data were collected through an online survey distributed via social media.

**Results:**

Among 402 participants, 57.2% had heard of the shingles vaccine, but only 7.7% received it. However, 53.2% expressed willingness to be vaccinated. Multivariable analysis revealed that those aged 56–60 were 1.8 times more likely to accept the vaccine than those aged 50–55 years (*p* = 0.03). Men were 1.9 times more likely to accept the vaccine than women (*p* = 0.01). Additionally, participants with a primary education were 16.1 times more likely to accept the vaccine than those with a higher education (p = 0.01).

**Conclusion:**

This study highlights the need for increased awareness and education among healthcare providers and the public in Saudi Arabia regarding shingles and its vaccine. The low vaccine uptake calls for effective strategies, such as awareness campaigns and provider reminders. Primary education and vaccine hesitancy influence willingness to be vaccinated.

## Plain Language Summary

This study aimed to understand how much people in Saudi Arabia know about shingles (herpes zoster) and the vaccine to prevent it. Shingles is a viral infection caused by the same virus that causes chickenpox. After having chickenpox, the virus can stay in the body and reactivate later in life, causing shingles. We conducted an online survey among adults aged 50 years and above to gather information. Many people had heard about shingles, but their knowledge about it was limited. Healthcare providers were not a significant source of information for most participants, showing the need for more awareness and education from healthcare professionals. We also looked at people’s attitudes towards the shingles vaccine. Only a small percentage of participants had received the vaccine, but many expressed willingness to get vaccinated. However, certain factors like age, gender, and education level influenced people’s willingness to take the vaccine. The low vaccine uptake is concerning because shingles can lead to serious health problems. To increase vaccine acceptance, we suggest implementing awareness campaigns and providing reminders to people and healthcare providers. It’s also important to address concerns about vaccine safety and efficacy. In conclusion, our study showed the need for more awareness and education about shingles and its vaccine among adults in Saudi Arabia. By addressing the barriers to vaccine uptake and promoting its benefits, we can reduce the burden of shingles and its complications in the older population.

## Background

Herpes zoster, commonly known as shingles, is a viral infection caused by reactivation of the varicella-zoster virus, which also causes chickenpox. After a person recovers from chickenpox, the virus can remain dormant in the body and reactivate later in life, leading to shingles [1]. The prevalence of herpes zoster in Saudi Arabia is unclear; however, its incidence is increasing globally, particularly in the elderly population [2]. Shingles can lead to serious complications, including post-herpetic neuralgia, vision loss, and neurological problems [3].

Herpes zoster vaccine is a safe and effective way to prevent shingles and complications. The vaccine is recommended for individuals aged ≥ 50 years, and a two-dose schedule is recommended for optimal protection [4]. In Saudi Arabia, the herpes zoster vaccine is available free of charge for individuals aged 50 years and above.

Several sociodemographic factors may influence the awareness and uptake of the herpes zoster vaccine, including age, sex, education level, income, and access to healthcare services. Older individuals and those with limited access to healthcare services may have lower awareness and uptake [5]. Cultural and religious beliefs may also influence vaccine acceptance in some populations, highlighting the need for culturally sensitive interventions to increase vaccination coverage [6, 7].

Despite the availability of effective vaccines, herpes zoster vaccination rates remain suboptimal in many countries, including Saudi Arabia [5, 8]. Studies have shown that various factors influence vaccine uptake, including sociodemographic factors, such as age, gender, education level, income, and access to healthcare services. Cultural and religious beliefs may also influence vaccine acceptance. In Saudi Arabia, limited studies have examined the practices related to the herpes zoster vaccine, with one recent study finding that only 4.5% of adults had received the vaccine [8]. Previous studies have focused on specific geographic areas or risk groups [9, 10], highlighting the need for a more comprehensive understanding of the population’s knowledge and attitudes towards shingles and its vaccine in Saudi Arabia. Increasing vaccination rates is crucial for reducing the burden of herpes zoster and its complications, particularly in the elderly population.

## Methods

### Study design

This study employed a cross-sectional design, as it was suitable for examining the knowledge and awareness of herpes zoster and its vaccine among the Saudi Arabian population.

### Study population

The study population included individuals aged ≥ 50 years who were currently residing in Saudi Arabia. This age range was chosen as it is the recommended age group for herpes zoster vaccination, and this population is at a higher risk of developing the disease.

### Survey questionnaire

The questionnaire used in this study was adapted from a previous study [7], and modifications were made to suit the study’s objectives and population. It comprises four sections. The first section gathered demographic information from the participants, such as age, gender, occupation, nationality, place of residence, educational level, and chronic diseases. The second section assessed the participants’ knowledge of shingles, including their sources of information, perceived risk factors, symptoms, signs, and complications of shingles, and misconceptions. The third section evaluated participants’ knowledge of the shingle vaccine, including their awareness of the vaccine, sources of information, target group of the vaccine, and misconceptions. The fourth section explored participants’ attitudes towards shingles and their vaccine, including their willingness to take the vaccine and barriers to receiving it.

### Data collection

To ensure that the sample was representative of the population, the survey was distributed through social media platforms such as Facebook, Twitter, and WhatsApp, which are widely used by people of interest. Data were collected through an online survey using the Google Forms platform between January and February 2023. The survey link was distributed through social media platforms. Participants who met the eligibility criteria were directed to the online survey, which took approximately 10–15 min to complete. The survey was available in Arabic and English to accommodate the participants’ preferences. To avoid duplicate responses, each participant completed the survey only once.

### Data analysis

The collected data were analyzed using Statistical Package for the Social Sciences (SPSS) version 28.0. Descriptive statistics, such as frequency, percentages, means, and standard deviations, were used to summarize the participants’ demographic characteristics, knowledge, and attitudes towards shingles and their vaccines. Bivariate analysis was conducted using the chi-squared test to examine the association between demographic characteristics and willingness to receive vaccination. Multivariable analysis was then conducted to identify the independent factors associated with the willingness to be vaccinated. Statistical significance was set at P < 0.05.

### Ethical considerations

This study was approved by the Institutional Review Board, and informed consent was obtained from all participants before they completed the questionnaire. Confidentiality and anonymity of participants were ensured throughout the study.

## Results

### Demographic characteristics

The study included 402 participants aged 50 years and above, with a higher proportion of females (67.9%) than males (32.1%). More than half of the participants (54.5%) were aged 50–55 years, followed by those aged 56–60 years (27.1%). Most participants had a higher education level (65.7%) and the majority were from Saudi Arabia (97.8%). Among the employed participants, 173 (43.0%) were in the teaching and education sector. The most common chronic conditions reported were hypertension (29.9%), diabetes mellitus (29.4%), and dyslipidemia (25.1%) (Table 1).

**Table 1:** Demographic Characteristics of the Study Population

Variable		n	(%)
Age Group (years)	50–55	219	(54.5)
	56–60	109	(27.1)
	61–65	47	(11.7)
	> 65	27	(6.7)
Gender	Male	129	(32.1)
	Female	273	(67.9)
Employment Status	Employed	130	(32.3)
	Self-Employed	18	(4.5)
	Retired	168	(41.8)
	Unemployed	86	(21.4)
Employment Area	Teaching & Education	173	(43.0)
	Engineering & Technology	11	(2.7)
	Arts & Communication	10	(2.5)
	Healthcare & Medicine	12	(3.0)
	Public Service & Administration	73	(18.2)
	Not Applicable	92	(22.9)
	Other	31	(7.7)
Country of Origin	Saudi	393	(97.8)
	Non-Saudi	9	(2.2)
Residential Location	Central Region	80	(19.9)
	Eastern Region	117	(29.1)
	Northern Region	43	(10.7)
	Southern Region	58	(14.4)
	Western Region	104	(25.9)
Healthcare Coverage	No	235	(58.5)
	Yes	167	(41.5)
Educational Level	Primary Education	17	(4.2)
	Middle School	28	(7.0)
	High School	93	(23.1)
	Higher Education	264	(65.7)
Chronic Conditions	Hypertension	120	(29.9)
	Diabetes Mellitus	118	(29.4)
	Dyslipidemia	101	(25.1)
	Coronary Artery Disease	8	(2.0)
	Asthma	31	(7.7)
	Hypothyroidism	45	(11.2)
	Gout	23	(5.7)
	Rheumatoid Arthritis	21	(5.2)
	Osteoarthritis	10	(2.5)
	Depression	16	(4.0)

### Knowledge of shingles

Overall, 328 (81.6%) participants reported having heard of shingles. Over one-third of the participants (n = 141, 35.1%) learned about shingles from their family or friends, followed by knowing someone who had shingles (n = 136, 33.8%). Healthcare providers were the source of information for only 30 (7.5%) participants, while the Internet, including social media and websites, was the source of information for 91 (22.6%) participants. Additionally, only 18 (4.5%) participants had personal experiences with shingles (Table 2).

**Table 2:** Survey Responses on Sources of Information about Shingles and Its Vaccine

Question		n	(%)
**How did you learn about the shingles?**	Healthcare provider	30	(7.5)
	Family or friends	141	(35.1)
	The internet (e.g., social media, websites)	91	(22.6)
	Personal experience of having shingles	18	(4.5)
	Knowing someone who had shingles	136	(33.8)
	Other	34	(8.5)
**How did you learn about the shingles vaccine?**	Healthcare provider	174	(43.3)
	Family or friends	139	(34.6)
	Someone who had shingles	187	(46.5)
	Vaccination schedule	206	(51.2)
	The internet (e.g., social media, websites)	125	(31.1)
	Other	202	(50.2)

Of the participants, 64 (15.9%) reported having chickenpox. Additionally, 72 participants (17.9%) believed that they had chickenpox before putting them at a higher risk of getting shingles, while 99 participants (24.6%) did not believe that they could get shingles if they came into contact with someone who had it. Furthermore, only 32 participants (8%) correctly identified that there was no cure for the shingles.

Table 3 summarizes the participants’ responses to questions about risk factors, susceptible groups, and the signs, symptoms, and complications of shingles. The most commonly identified perceived risk factor for shingles was a weakened immune system (n = 133, 33.1%), followed by advanced age (n = 85, 21.1%) and chronic diseases (n = 74, 18.4%). Nearly half of the participants correctly identified the elderly as the group most susceptible to shingles (n = 193, 48%), whereas only a small percentage identified pregnant women (n = 23, 5.7%). The most commonly identified sign or symptom of shingles was rash (n = 250, 62.2%) followed by blisters (n = 139, 34.6%). However, fever was incorrectly reported as a symptom by 142 (35.3%) participants.

**Table 3:** Survey Responses on Knowledge of Shingles

Question		n	(%)
**What do you think to be risk factors for developing shingles?**	Age	85	(21.1)
	Gender	25	(6.2)
	Chronic diseases	74	(18.4)
	Weakened immune system	133	(33.1)
	High levels of stress	53	(13.2)
	Poor quality sleep	21	(5.2)
	Poor diet	51	(12.7)
	Mobile phone usage	5	(1.2)
**Which groups of people are susceptible to shingles?**	Children	21	(5.2)
	Elderly	193	(48.0)
	Males	34	(8.5)
	Females	42	(10.4)
	Immunocompromised	111	(27.6)
	Pregnant Women	23	(5.7)
	All age groups	58	(14.4)
**What are the signs, symptoms, and complications of shingles?**	Rash	250	(62.2)
	Blisters	139	(34.6)
	Cough	67	(16.7)
	Fever	142	(35.3)
	Sore Throat	21	(5.2)
	Neuropathic Pain	83	(20.6)
	Blindness	24	(6.0)

### Attitudes toward shingles

**Fig. 1:**
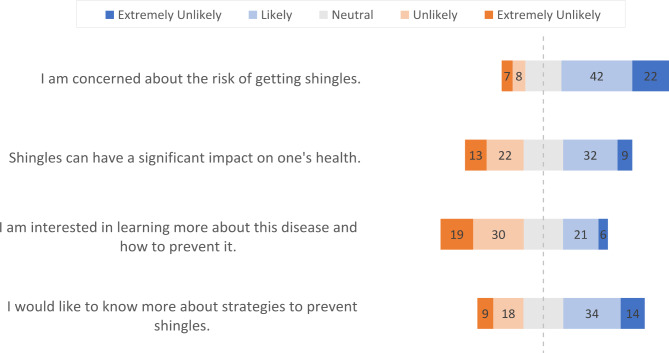
Survey responses on attitudes toward shingles

Figure 1 shows that 133 (33.0%) participants reported being concerned about getting shingles and 253 (62.9%) reported that shingles can have a significant impact on their health. The majority of participants (n = 319, 79.4%) were interested in learning more about shingles and how to prevent them, and 343 (85.3%) reported that they would like to know more about the strategies to prevent shingles.

### Knowledge of shingles vaccine

Overall, 230 (57.2%) participants reported having heard of the Shingle vaccine. The primary sources of information were the vaccination schedule (n = 206, 51.2%) and knowing someone who had shingles (n = 187, 46.5%), while healthcare providers were the primary sources for 174 (43.3%) participants. Only 108 (26.9%) participants correctly identified that the recommended age for receiving the vaccine was over 50 years.

### Attitudes toward shingles vaccine

Only 31 (7.7%) patients reported receiving the Shingle vaccine. However, 214 (53.2%) participants expressed willingness to receive the vaccine. Statistically significant differences in the willingness to take the shingle vaccine were observed based on age group, residential location, and educational level (Table 4). Multivariable logistic regression analysis showed that participants in the 56–60 age group were 1.8 times more likely to be willing to take the vaccine than those in the 50–55 age group (p = 0.03). Men were 1.9 times more likely to be willing to take the vaccine than women (p = 0.01). Furthermore, participants from the Southern Region were 2.3 times more likely to be willing to take the vaccine than those from the Western Region (p = 0.02). Finally, participants with a primary education were 16.1 times more likely to be willing to take the vaccine than those with a higher education (p = 0.01) (Table 5).

**Table 4:** Willingness to Take Shingles Vaccine by Demographic Factors

Variable		n	(%)	*P* value
Age Group (years)	50–55	111	(50.7)	**0.02**
	56–60	71	(65.1)	
	61–65	20	(42.6)	
	> 65	12	(44.4)	
Gender	Male	77	(59.7)	0.08
	Female	137	(50.2)	
Employment Status	Employed	76	(58.5)	0.48
	Self-Employed	10	(55.6)	
	Retired	83	(49.4)	
	Unemployed	45	(52.3)	
Employment Area	Teaching & Education	90	(52.0)	0.71
	Engineering & Technology	5	(45.5)	
	Arts & Communication	4	(40.0)	
	Healthcare & Medicine	9	(75.0)	
	Public Service & Administration	38	(52.1)	
	Not Applicable	50	(54.3)	
	Other	18	(58.1)	
Country of Origin	Saudi	208	(52.9)	0.41
	Non-Saudi	6	(66.7)	
Residential Location	Central Region	45	(56.3)	**0.02**
	Eastern Region	52	(44.4)	
	Northern Region	27	(62.8)	
	Southern Region	40	(69.0)	
	Western Region	50	(48.1)	
Healthcare Coverage	No	124	(52.8)	0.82
	Yes	90	(53.9)	
Educational Level	Primary Education	16	(94.1)	**0.01**
	Middle School	14	(50.0)	
	High School	52	(55.9)	
	Higher Education	132	(50.0)	

**Table 5:** Multivariable Logistic Regression Analysis of Factors Associated with the Willingness to Take the Shingles Vaccine

Variable		OR	(95% CI)	*P* value
Age Group (years)	50–55	Reference Group		
	56–60	1.8	(1.1–3.0)	**0.03**
	61–65	0.7	(0.3–1.4)	0.29
	> 65	0.6	(0.2–1.6)	0.31
Gender	Male	1.9	(1.2–3.1)	**0.01**
	Female	Reference Group		
Residential Location	Central Region	1.3	(0.7–2.3)	0.48
	Eastern Region	0.9	(0.5–1.6)	0.64
	Northern Region	1.7	(0.8–3.6)	0.18
	Southern Region	2.3	(1.1–4.7)	**0.02**
	Western Region	Reference Group		
Educational Level	Primary Education	16.1	(2.0–132.0)	**0.01**
	Middle School	0.9	(0.4–2.0)	0.73
	High School	1.2	(0.7–2.0)	0.50
	Higher Education	Reference Group		

### Barriers to receiving the shingles vaccine

**Fig. 2:**
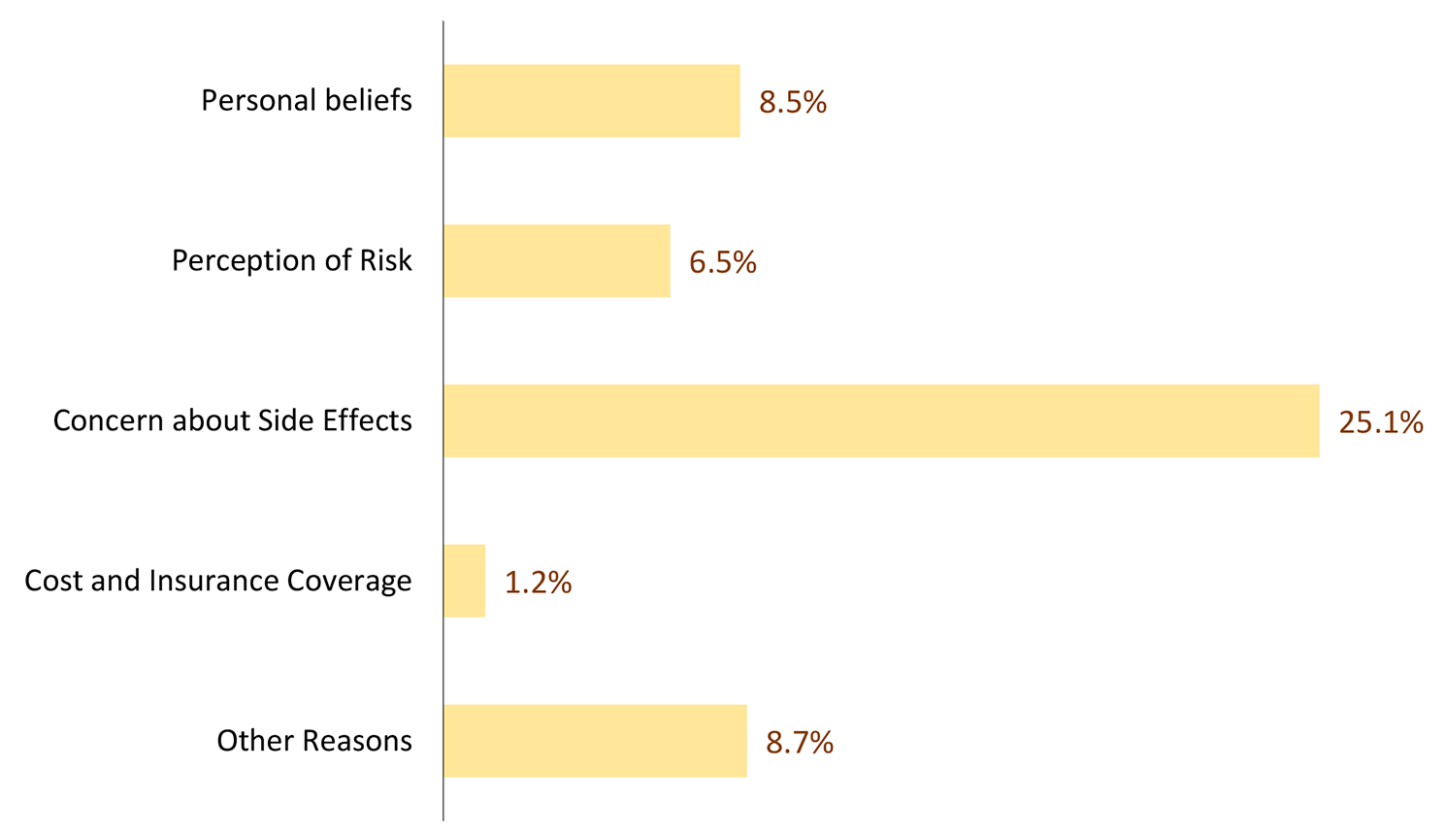
Barriers to receiving the shingles vaccine

Figure 2 summarizes the barriers that prevented the participants from receiving the shingle vaccine. Of the participants, 101 (25.1%) reported concerns about potential side effects, while 34 (8.5%) cited personal beliefs as barriers, such as not believing in vaccines or preferring to take medicine when sick. In addition, 26 (6.5%) participants did not perceive themselves to be at risk or did not know that the vaccine existed. Other barriers included cost and insurance coverage (n = 5, 1.2%), and miscellaneous reasons (n = 35, 8.7%).

## Discussion

This study aimed to assess the knowledge, attitudes, and barriers towards shingles and its vaccine among participants aged 50 years and above in Saudi Arabia. The findings of the study revealed that, although the majority of participants had heard of shingles before, healthcare providers were not a significant source of information. This highlights the need for increased awareness and education regarding shingles by healthcare providers in Saudi Arabia.

The participants in the study demonstrated a fair understanding of the risk factors, susceptible groups, signs, symptoms, and complications of shingles. However, misconceptions about the symptoms and cure of shingles are evident. For instance, 35.3% of participants incorrectly reported fever as a symptom. These findings underscore the importance of patient education in dispelling common misconceptions about the disease and its vaccines.

The study’s findings also highlight the need to increase awareness about the shingle vaccine, as only 57.2% of the participants had heard of it. However, over half of the participants expressed a willingness to receive the vaccine, suggesting the potential for increasing vaccine uptake through targeted educational initiatives. Healthcare providers can play an essential role in promoting vaccine uptake as they are the primary source of information about the vaccine for over 40% of the participants. Previous research has shown that education and awareness campaigns can be effective in increasing vaccine uptake among older adults [11, 12]. Therefore, targeted campaigns should be developed and implemented to increase the awareness of shingles and their vaccines among healthcare providers and the general population.

The low uptake of the shingle vaccine (7.7%) among the study samples is concerning. Despite the availability of the vaccine, many individuals may not perceive themselves to be at a high risk of developing shingles or may be hesitant to receive the vaccine due to concerns about safety or efficacy. However, shingles can cause significant morbidity, including postherpetic neuralgia, which can result in chronic pain and a decreased quality of life. Therefore, efforts to increase vaccine uptake are crucial to prevent shingles and complications. Strategies such as education and awareness campaigns, provider reminders, and reducing vaccine cost barriers have been effective in increasing vaccination rates for other diseases, and could be applied to increase vaccine uptake by shingles [11, 12].

The unexpected finding that participants with primary education were more willing to take the shingles vaccine than those with higher education raises questions about the role of health literacy and vaccine hesitancy in vaccine uptake. Previous studies have found that individuals with lower educational levels are often at a disadvantage in terms of health literacy, which may impact their ability to understand and act on health-related information, including recommendations for vaccination [13, 14]. However, some studies have reported that higher education levels may be associated with increased vaccine hesitancy, which is defined as a delay or refusal of vaccination despite the availability of vaccine services [15]. However, other studies have found no association between education level and vaccine hesitancy education level and vaccine hesitancy [16]. One possible explanation for the observed association between primary education and willingness to receive the shingle vaccine is that individuals with lower education levels may have less access to healthcare services and, therefore, may be more motivated to take advantage of preventive health measures when they become available. Additionally, people with lower educational levels may have higher levels of trust in healthcare providers and are more likely to follow their recommendations [17].

This study has several limitations that should be considered when interpreting the results. The study relied on self-reported data, which may have been subject to recall bias or social desirability bias. This study did not explore the impact of cultural or social factors on vaccine acceptance, which could be particularly relevant in the Saudi Arabian context. Despite these limitations, the findings of this study provide valuable insights into the knowledge, attitudes, and barriers towards shingles and their vaccination among older adults in Saudi Arabia. They also highlight the need for targeted educational interventions to improve vaccine uptake and prevent shingle-related morbidity.

## Conclusion

The study sheds light on the knowledge, attitudes, and obstacles faced by older adults in Saudi Arabia regarding shingles and their vaccines. These findings underscore the crucial need for increased awareness and education among the general public about shingles and the benefits of vaccination. The low uptake of the shingle vaccine among our study participants is a cause for concern and calls for the implementation of effective strategies such as awareness campaigns, provider reminders, and reducing vaccine cost barriers to increase vaccine uptake. Moreover, the unexpected finding that participants with primary education were more willing to take the shingle vaccine than those with higher education highlights the role of health literacy and vaccine hesitancy in vaccine uptake. Overall, this study highlights the importance of targeted educational interventions to improve vaccine uptake and prevent shingle-related morbidity in older adults in Saudi Arabia.

## Data Availability

The datasets generated and/or analyzed during the current study are available from the corresponding author upon reasonable request.
